# Correlation between degeneration of cervical intervertebral disc and degeneration of paravertebral muscle

**DOI:** 10.3389/fendo.2024.1391970

**Published:** 2024-06-19

**Authors:** Qiujiang Li, Xingxia Long, Rui Wang, Pengying Niu, Lijun Cai, Lei Wang, Yueming Song

**Affiliations:** ^1^ Department of Orthopaedics, Orthopaedic Research Institute, West China Hospital, Sichuan University, Chengdu, China; ^2^ Department of Thoracic Surgery, West China Hospital, Sichuan University/West China School of Nursing, Sichuan University, Chengdu, China; ^3^ Medical Center, People’s Hospital of Ningxia Hui Autonomous Region, Yinchuan, Ningxia, China; ^4^ Department of Orthopedics, People’s Hospital of Ningxia Hui Autonomous Region, Yinchuan, Ningxia, China

**Keywords:** paraspinal muscle degeneration, intervertebral disc degeneration, mDIXON-Quant, fat infiltration, cervical

## Abstract

**Objective:**

To investigate the relationship between degeneration of cervical intervertebral disc and degeneration of paravertebral muscles[multifidus (MF), cervical semispinalis (SCer), semispinalis capitis (SCap) and splenius capitis (SPL)].

**Methods:**

82 patients with chronic neck pain were randomly selected, including 43 males and 39 females, with 50.73 0.7.51 years old. All patients were scanned by 3.0T MRI Philips Ingenia performed conventional MRI sequence scanning and fat measurement sequence mDIXON-Quant scanning of cervical. Fat infiltration (FI) and cross-sectional area (CSA) of cervical paravertebral muscle (MF, SCer, SCap and SPL) at central level of C5–6 disc were measured by Philips 3.0T MRI image post-processing workstation. According to Pfirrmann grading system, there was no grade I in the included cases. The number of grade IIr IV cases were n=16, 40, 19 and 7 respectively. CSA and FI of cervical paravertebral muscles were compared with t test or one-way ANOVA, Spearman correlation analysis was used to evaluate the correlation between age, disc degeneration, and CSA, FI of cervical paravertebral muscles, and multiple linear regression analysis was used to analyze the independent influencing factors of CSA and FI.

**Results:**

CSA of cervical paravertebral muscles in male patients was significantly higher than that in female patients (all P<0.001), but there was no significant difference in FI (all P>0.05). Age was weakly correlated with CSA of MF+SCer, moderately correlated with CSA of SCap and SPL (r=-0.256, -0.355 and -0.361, P<0.05), weakly correlated with FI of SCap and SPL (r= 0.182 and 0.264, P<0.001), moderately correlated with FI of MF+SCer (r=0.408, P<0.001). There were significant differences in FI with disc degeneration (P<0.001, P=0.028 and P=0.005). Further correlation analysis showed that disc degeneration was strongly correlated with FI of MF+SCer (r=0.629, P<0.001), and moderately correlated with FI of SCap and SPL (r=0.363, P=0.001; r=0.345, P=0.002). Multiple linear regression analysis showed that sex and age were the influencing factors of CSA of SCap and SPL, sex was the independent influencing factor of CSA of MF+SCer, and disc degeneration was the independent influencing factor of FI.

**Conclusions:**

Age is negatively correlated with CSA and positively correlated with FI. Disc degeneration was correlated with FI of paravertebral muscles, especially with FI of MF and SCer. Sex and age were the influencing factors of CSA, while disc degeneration was the independent influencing factor of FI.

## Introduction

Chronic neck pain and low back pain are common symptoms in the general population, mostly in middle-aged and elderly people, and increase with age (1). The degeneration of paravertebral muscles may be one of the important causes of chronic neck pain and low back pain (2). One study has reported reduced muscle CSA in patients with low back pain compared to asymptomatic controls (3). Similarly, related studies have found that paraspinal muscle atrophy is more pronounced in patients with low back pain than in those without low back pain, and that paraspinal muscle atrophy is more pronounced on the symptomatic side than on the asymptomatic side (4–6). Therefore, many studies have confirmed that lumbar paravertebral muscle degeneration is closely related to the occurrence, development, clinical efficacy and prognosis of lumbar degenerative diseases. Paracervical musculature plays an important role in maintaining the level and neutrality of the cervical spine and in distributing head loads through flexion, extension, and translation movements (7, 8). It is estimated that neck muscles provide about 80% of total stability, while bony ligament structures contribute the remaining 20%. Degeneration of the deep neck extensors, which support neck movement and provide neck stability, has been associated with neck pain.

Spinal degeneration begins at the intervertebral disc. The early imaging signs of cervical disc degeneration show changes in disc signal, but few scholars have evaluated the FI of paravertebral muscles and cervical disc degeneration. Therefore, the correlation between disc signal and paravertebral muscle degeneration can better reflect the role of paravertebral muscle in the occurrence and development of cervical degenerative diseases. However, there are no relevant research reports. Meanwhile, most of the studies are subjective evaluation or semi-quantitative analysis of paravertebral muscle degeneration, and there are observation data bias and lack of reliability of experimental data to some extent (9, 10). The mDIXON-Quant technology is a new magnetic resonance scanning technology for quantitative fat measurement based on the principle of water-fat separation technology based on chemical shift introduced in recent years (11–13). At present, it is mainly applied to quantitative measurement of liver fat, but there is no report on FI evaluation of paravertebral muscle (11). Therefore, this study intends to use mDIXON-Quant technique to quantitatively measure FI of paravertebral muscles and evaluate the correlation between FI of paravertebral muscles and cervical disc degeneration.

## Methods

### Patient population

82 patients with chronic neck pain were randomly selected from outpatient or inpatient department. In order to exclude the influence of different MRI machines on the results, we uniformly selected the cases scanned by Philips Ingenia 3.0T MRI scanner in our hospital. Inclusion criteria were as follows: (1) Patients with chronic neck pain; (2) mDIXON-Quant sequence for 3.0T MRI and fat measurement; (3) aged from 30 to 75 years old; (4) Body Mass Index (BMI) between 18.5 and 23.9; (5) No physical therapy, acupuncture and other treatment measures affecting paravertebral muscles. Exclusion criteria were as follows: (1) Patients with a history of cervical spine surgery or cervical spine trauma; (2) Patients with concomitant diseases of important organs or serious systemic diseases; (3) MRI images were poor, and the range and boundary of muscles could not be distinguished and recognized well;④ Patients with MRI contraindications exist.

### MRI scanning methods

Conventional MRI sequences (T1WI, T2WI, T2WI-mDIXON) and fat-measuring sequences (mDIXON-Quant) of cervical spine were performed with Philips Ingenia in supine position. mDIXON-Quant parameters: flip angle, 3°; TR, 5.8 ms; TE, 1.02 ms; voxel, 2.5 mm×2.5 mm×6mm; slice thickness, 6mm; field of view, 400mm×350mm×210mm; matrix, 160×40×70; and number of excitations, 1. The relevant scanning parameters are shown in **Table 1**.

**Table 1:** List of scanning parameters of MRI sequences.

Scan parameters	T1WI	T2WI	T2WI-mDIXON	mDIXON-Quant
TR	454	2500	2500	5.8
TE	8	110	100	1.02
FOV	160×254	200×162	160×250	400×350
SNR	1	1.8	1	1
NSA	1	1	1	1
Layer thickness/spacing	3.0/0.3	3.0/0.3	3.0/0.3	6.0/-3
scanning time, s	2:00	2:25	2:25	0:14

### Pfirrmann grade

We graded the degree of cervical disc degeneration by using the internationally common Pfirrmann classification system on the median sagittal position of 3.0T MRI with the help of Picture Archiving and Communications Systems (PACS) (14). Pfirrmann classification system divides disc degeneration into 5 grades. Pfirrmann grade I is mainly found in children, so only Pfirrmann grade II~IV disc degeneration is studied in this study. C5–6 is the most common segment for cervical disc degenerative disease, so this study will analyze this segment. The cervical disc grading results were assessed independently by 3 spine surgeons, and the results with different opinions were discussed and decided together. Finally, all the results were summarized and collected.

### CSA and FI determination of cervical paravertebral muscles

**Figure 1:**
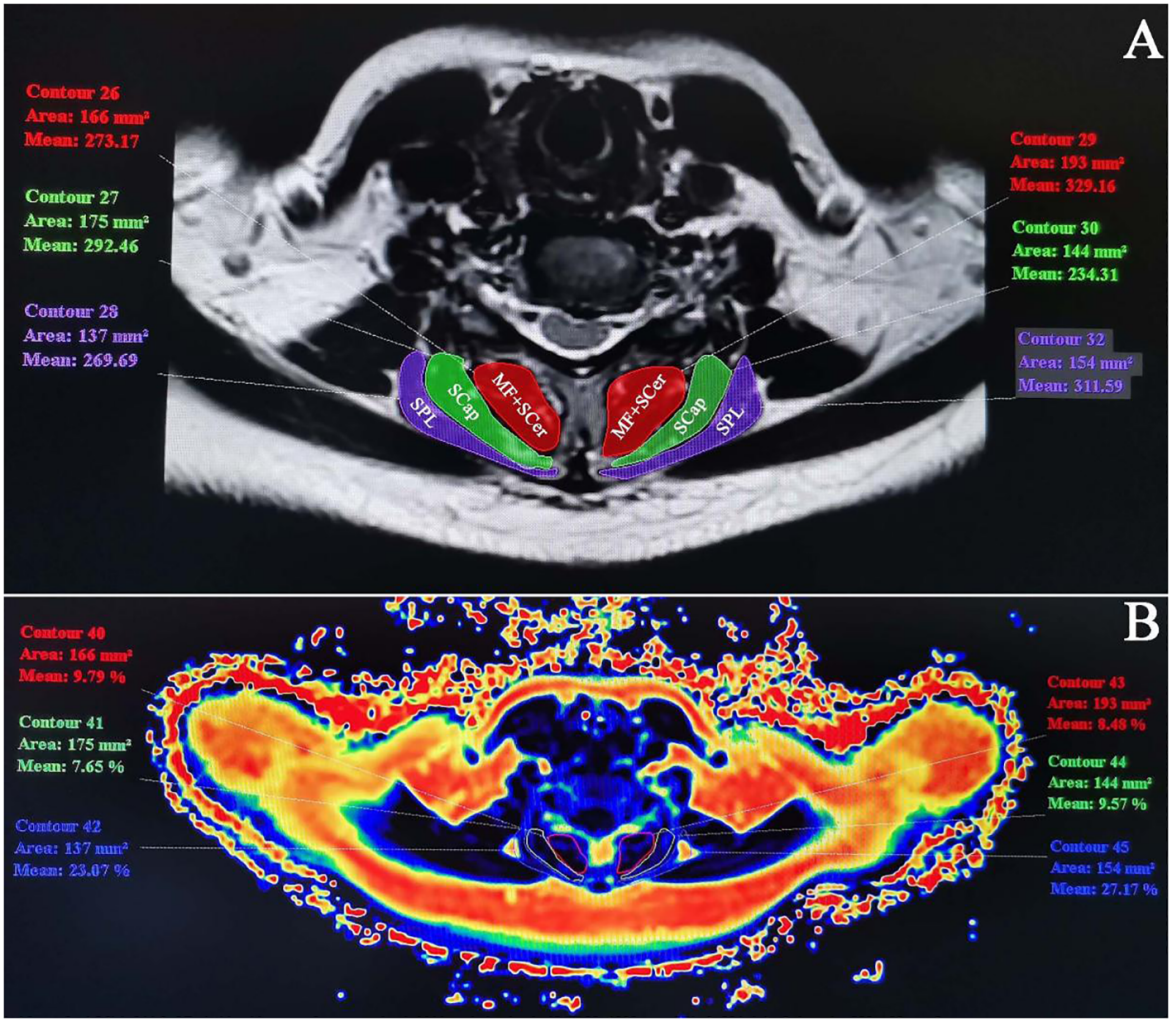
CSA and FI measurements of cervical paravertebral muscles at C5–6. **(A)** C5–6 T2WI axial sequence. CSA was measured along the outline of the muscles: MF+SCer, SCap and SPL. **(B)** Fat pseudo-color map. Measure ROI muscle FI in FF image.).

The axial scan images of mDIXON-Quant sequence were transmitted to Philips 3.0T MRI image post-processing workstation. Two doctors with more than 5 years of experience in skeletal muscle imaging delineated the region of interest (ROI) of all C5–6 disc center layers on fat fraction images respectively. The ROI of cervical paravertebral muscles was set at multifidus (MF), cervical semispinalis (SCer), semispinalis capitis (SCap) and splenius capitis (SPL) on both sides respectively. Along the muscle contour, the system automatically generates ROI fat fraction (FF), i.e. FI and CSA, as shown in **Figure 1**. The left and right sides of each paravertebral muscle were measured three times and the average value was taken. The CSA representative value of the muscle at the same level was taken as the average value of CSA of the muscle at the same level. Similarly, the average FI value of the left and right muscles at the same level was taken as the FI representative value of the muscle at that level. In previous studies, MF and SCer muscles were not clearly demarcated on MRI in some cases, and both muscles belonged to deep neck extensors. Therefore, MF and SCer muscles were analyzed as a whole in this study.

### Consistency evaluation of CSA and FI measurements

Twenty patients were randomly selected for repeated measurement. CSA and FI of paracervical muscles were measured independently and without interference by two observers. Inter-observer consistency of measurements was evaluated using intra-class correlation coefficients (ICC) between two observers measuring one week apart; in addition, intra-observer consistency of measurements was evaluated using ICC when one of the observers repeated measurements one week later. ICC>0.75 is considered to have good measurement consistency.

### Statistical analysis

Data were analyzed statistically using SPSS 26.0 software (SPSS Inc., Chicago, IL, USA) and presented as mean ± standard deviation. For quantitative data conforming to normal distribution, t test was used for comparison between two groups, variance analysis was used for comparison between multiple groups, and rank sum test was used for non-normal distribution. Spearman correlation analysis was used to analyze the correlation between variables, and multiple linear regression analysis was used to analyze the independent influencing factors of paravertebral muscle degeneration. Correlation coefficient r≥0.7 was considered significant correlation; r=0.5~0.7 was strong correlation; r=0.3~0.5 was moderate correlation; r ≤ 0.3 was weak correlation. P<0.05 was statistically significant.

## Results

The ICC of CSA and FI of cervical paraspinal muscles was greater than 0.75 within and between observers, indicating good consistency (**Table 2**). A total of 82 patients were included in this study, 43 males and 39 females, with an average age of 50.73 0.7.51 years, of which 40 - 50 years and 50 - 60 years were the main population, accounting for 30.49% (25/82) and 35.37% (29/82) respectively. In this study, C5–6 was selected as the most common cervical segment degeneration. 16 cases (19.51%), 40 cases (48.78%), 19 cases (23.17%) and 7 cases (8.54%) of C5–6 were grade II, III, IV and V, respectively (**Table 3**).

**Table 2:** Intra-and inter-observer consistency of CSA and FI measurements of cervical paravertebral muscles.

Measurement parameters	Intra-observer		Inter-observer	
	ICC	95% *CI*	ICC	95% *CI*
C5-6 CSA (MF+SCer)	0.907	0.784-0.962	0.905	0.751-0.963
C5-6 FI (MF+SCer)	0.932	0.267-0.983	0.925	0.822-0.970
C5-6 CSA (SCap)	0.863	0.686-0.944	0.876	0.714-0.949
C5-6 FI (SCap)	0.881	0.723-0.951	0.872	0.701-0.948
C5-6 CSA (SPL)	0.921	0.814-0.968	0.901	0.769-0.960
C5-6 FI (SPL)	0.852	0.665-0.939	0.802	0.570-0.916

**Table 3:** General information of patients with cervical degenerative diseases.

General information	Number of cases/average
Average age, years	50.73 ± 9.51
Age, years	
30~40	17 (20.73%)
40~50	25 (30.49%)
50~60	29 (35.37%)
>60	11 (13.41%)
Sex, n (%)	
Male	43 (52.44%)
Female	39 (47.56%)
Pfirrmann grade, n (%)	
II	16 (19.51%)
III	40 (48.78%)
IV	19 (23.17%)
V	7 (8.54%)

**Figure 2:**
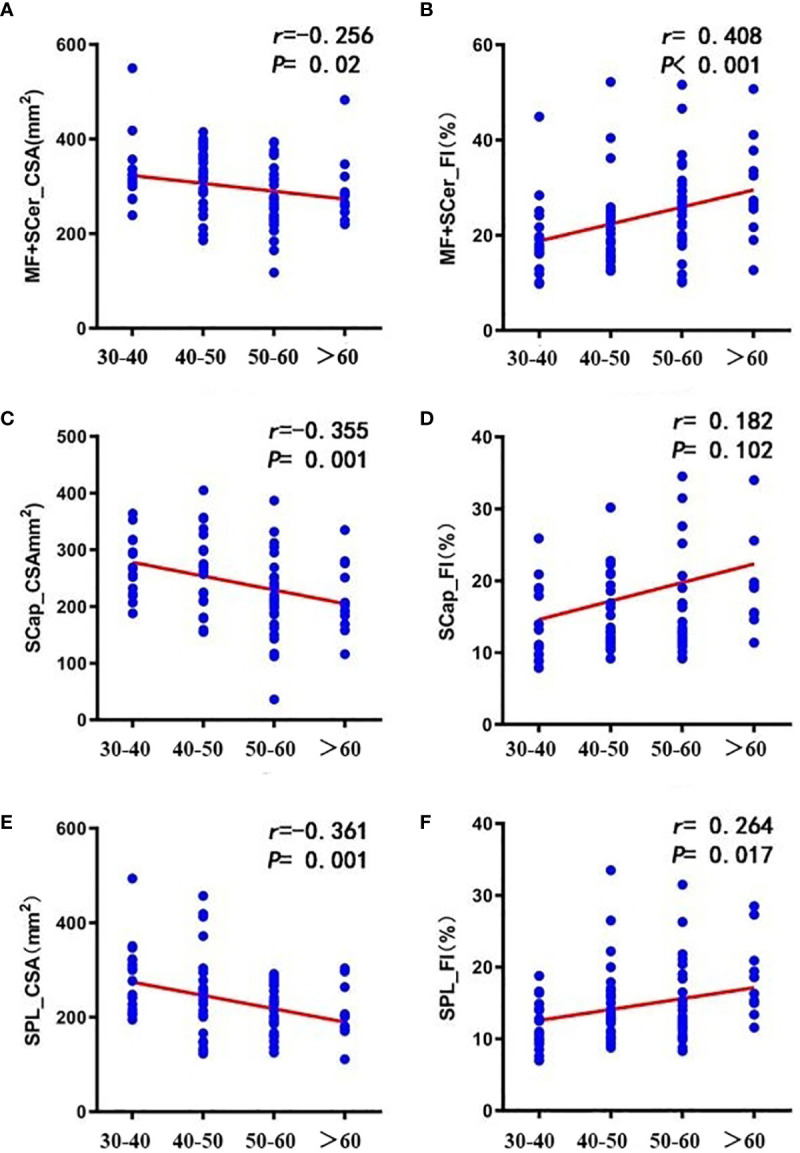
Correlation between age and CSA **(A, C, E)** and FI **(B, D, F)** of cervical paravertebral muscles. [**(A, B)** MF+SCer; **(C, D)** SCap; **(E, F)** SPL].

CSA in MF+SCer, SCap, and SPL were significantly higher in males than in females (all P<0.001), but not in FI (all P>0.05) (**Table 4**). In CSA of cervical paravertebral muscles, there were statistically significant differences in SCap and SPL between age (P=0.007 and P=0.005); in FI of cervical paravertebral muscles, there were statistically significant differences in MF+SCer and SPL between age (P=0.015 and P=0.019) (**Table 5**). Further Spearman correlation analysis showed that age was positively correlated with CSA of MF+SCer, SCap and SPL, and weakly correlated with CSA of MF+SCer, and moderately correlated with CSA of SCap and SPL (r =-0.256,-0.355 and-0.361, respectively, P<0.05); age was positively correlated with FI of MF+SCer, SCap and SPL, and weakly correlated with FI of SCap and SPL (r = 0.182 and 0.264, respectively), and moderately correlated with FI of MF+SCer (r=0.408, P<0.001) (**Figure 2**).

**Table 4:** Comparison of CSA and FI of Paracervical muscle between sex( $\bar{\text{x}}$ ± *s*).

Sex	Number	MF+SCer		SCap		SPL	
		CSA (mm^2^)	FI (%)	CSA (mm^2^)	FI (%)	CSA (mm^2^)	FI (%)
Male	43	338.35 ± 66.15	23.85 ± 10.14	283.67 ± 64.90	15.65 ± 5.62	278.70 ± 71.63	14.60 ± 5.28
Female	39	256.82 ± 48.45	23.82 ± 9.77	199.23 ± 44.37	15.99 ± 6.75	186.13 ± 44.95	14.89 ± 5.60
*T value*		6.312	0.015	6.808	-0.253	6.926	-0.239
*P value*		<0.001	0.988	<0.001	0.801	<0.001	0.812

**Table 5:** Comparison of CSA and FI of cervical paravertebral muscles between age ( $\bar{x}$ ± *s*).

Age	Number	MF+SCer		SCap		SPL	
		CSA (mm^2^)	FI (%)	CSA (mm^2^)	FI (%)	CSA (mm^2^)	FI (%)
30-40	17	323.00 ± 71.62	19.39 ± 8.32	270.88 ± 49.92	14.05 ± 5.40	281.00 ± 76.29	11.99 ± 3.41
40-50	25	315.16 ± 67.17	21.68 ± 9.36	267.96 ± 69.36	16.32 ± 5.84	246.68 ± 91.30	15.43 ± 5.58
50-60	29	274.93 ± 68.49	26.02 ± 9.62	217.03 ± 71.97	15.31 ± 6.62	206.03 ± 50.62	14.41 ± 5.52
>60	11	292.91 ± 73.32	29.84 ± 10.77	215.45 ± 63.66	18.70 ± 6.28	211.27 ± 57.18	18.29 ± 5.45
*F value*		2.320	3.684	4.322	1.414	4.571	3.527
*P value*		0.082	0.015	0.007	0.245	0.005	0.019

**Figure 3:**
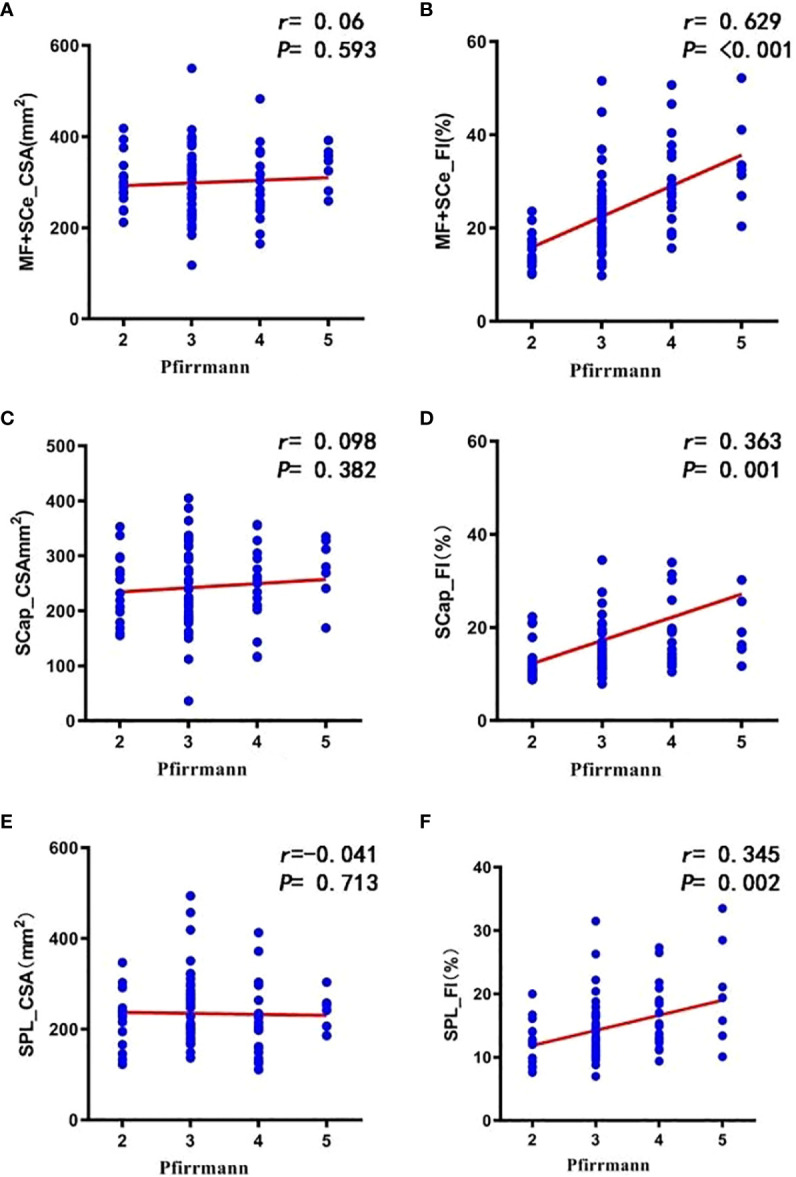
Correlation between the degree of disc degeneration and total CSA **(A, C, E)** and total FI **(B, D, F)** of cervical paravertebral muscles.

FI of cervical paravertebral muscles was significantly different in patients with different degree of disc degeneration (P<0.001, P=0.028 and P=0.005) (**Table 6**). Spearman correlation analysis showed that the degree of disc degeneration was strongly correlated with FI of MF+SCer (r=0.629, P<0.001), and moderately correlated with FI of SCap and SPL (r=0.363, P=0.001;r=0.345, P=0.002) (**Figure 3**).

**Table 6:** Differences in CSA and FI of cervical paravertebral muscles in patients with different degrees of cervical disc degeneration ( $\bar{x}$ ± *s*).

Pfirrmann grade	Number	MF+SCer		SCap		SPL	
		CSA (mm^2^)	FI (%)	CSA (mm^2^)	FI (%)	CSA (mm^2^)	FI (%)
II	16	303.31 ± 56.63	14.94 ± 4.02	241.75 ± 61.91	13.14 ± 4.68	224.06 ± 61.36	12.51 ± 3.53
III	40	293.65 ± 77.03	22.92 ± 8.45	239.33 ± 75.55	15.09 ± 5.63	246.78 ± 82.12	13.94 ± 4.90
IV	19	296.68 ± 76.82	29.51 ± 9.61	241.74 ± 70.30	18.37 ± 7.10	217.37 ± 83.34	16.26 ± 5.09
V	7	332.71 ± 47.79	34.00 ± 10.22	276.29 ± 58.05	19.10 ± 6.52	236.71 ± 40.02	20.26 ± 8.31
*F value*		0.617	12.886	0.556	3.198	0.766	4.699
*P value*		0.606	<0.001	0.645	0.028	0.517	0.005

Multivariate linear regression analysis was used to evaluate whether sex, age and disc degeneration grade were independent factors of CSA and FI. The results showed that sex and age were independent factors of CSA and SPL of paravertebral muscles; sex was independent factor of MF+SCer CSA; disc degeneration grade was independent factor of FI of paravertebral muscles (P<0.001, P<0.001, P=0.01, P=0.006) (**Tables 7**–**9**).

**Table 7:** Multiple linear regression analysis of independent influencing factors of MF+SCer CSA and FI in cervical paravertebral muscles.

	CSA					FI				
	B	SE	*β*	*t*	*P*	B	SE	*β*	*t*	*P*
Constant	410.86	32.554	–	12.621	<0.001	1.474	4.55	–	0.324	0.747
Sex	-76.272	13.592	-0.539	-5.612	<0.001	-0.145	1.9	-0.007	-0.076	0.939
Age	-9.357	7.688	-0.127	-1.217	0.227	1.488	1.075	0.145	1.384	0.17
Pfirrmann grade	7.438	8.426	0.09	0.883	0.38	5.918	1.178	0.512	5.026	<0.001

After multiple regression analysis by SPSS software, the standard coefficient of constant is –.

**Table 8:** Multiple linear regression analysis of independent influencing factors of CSA and FI in cervical paravertebral muscles.

	CSA					FI				
	B	SE	*β*	*t*	*P*	B	SE	*β*	*t*	*P*
Constant	354.826	30.034	–	11.814	<0.001	7.452	3.293	–	2.263	0.026
Sex	-73.472	12.54	-0.527	-5.859	<0.001	0.489	1.375	0.04	0.356	0.723
Age	-19.85	7.093	-0.274	-2.798	0.006	0.179	0.778	0.028	0.23	0.819
Pfirrmann grade	14.04	7.773	0.172	1.806	0.075	2.247	0.852	0.313	2.636	0.01

After multiple regression analysis by SPSS software, the standard coefficient of constant is –.

**Table 9:** Multivariate linear regression analysis of independent influencing factors of SPL CSA and FI in cervical paravertebral muscles.

	CSA					FI				
	B	SE	*β*	*t*	*P*	B	SE	*β*	*t*	*P*
Constant	391.838	32.758	–	11.962	<0.001	6.15	2.806	–	2.192	0.031
Sex	-83.368	13.677	-0.551	-6.096	<0.001	0.12	1.172	0.011	0.102	0.919
Age	-18.457	7.736	-0.235	-2.386	0.019	0.78	0.663	0.14	1.178	0.242
Pfirrmann grade	3.248	8.478	0.037	0.383	0.703	2.035	0.726	0.323	2.803	0.006

After multiple regression analysis by SPSS software, the standard coefficient of constant is –.

## Discussion

Cervical hypermobility and overload is an important factor causing cervical spine degeneration (15–17). Teraguchi et al. (18) surveyed 975 participants aged 21 - 97 years and found that the highest prevalence of disc degeneration was at the C5–6 level, 51.5% in males and 46% in females. Therefore, in this study, C5–6 segment with the most obvious and representative cervical degeneration was selected for the study of intervertebral disc degeneration. At present, a large number of studies have confirmed that the number of muscle fibers in muscle decreases gradually with the increase of age, and muscle fiber degeneration occurs gradually, resulting in muscle atrophy and mass decline (19). A 10-year MRI study of cervical posterior extensor CSA in asymptomatic subjects showed a gradual increase in muscle CSA in subjects aged 10 to 30 years and a gradual decrease in muscle CSA in subjects aged 40 years and older (11). Therefore, the study controlled the age of the population to be included before the reduction of muscle CSA, i.e., 30 years. Valera-Calero et al. (20) analyzed cervical extensor CSA in healthy subjects using panoramic ultrasound and found that males had greater cervical extensor CSA than females. Sasaki et al. (21) reported that CSA in men was greater than that in women, while FI in paravertebral muscles in women was higher than that in men. The results of our study showed that at C5–6 level, CSA of paracervical muscles in male patients was significantly greater than that in female patients. This result is consistent with the study described above.

At present, the evaluation of paravertebral muscle degeneration is divided into quantitative evaluation and visual semi-quantitative evaluation (9, 10). The most widely used semi-quantitative assessment is the Goutallier grading system (22, 23). However, visual semi-quantitative assessment methods are affected by inter-observer differences, which will affect the results of analysis to some extent. The mDIXON-Quant sequence is a 3-dimensional Fast Field Echo (3D-FFE) sequence that uses multiple acquired echoes to generate water, fat, in-phase, and inverted images synthesized from water-fat images (24–26). Because there is almost no limit on echo time, mDIXON-Quant has the advantage of being more efficient and accurate than other MR fat quantification techniques. However, only a few studies have reported quantitative measurements of paravertebral muscle fat based on the mDIXON-Quant sequence. Zhang et al. (27) evaluated the reliability of measuring fat content in lumbar bone marrow and paravertebral muscle using mDIXON-Quant sequence, and found that mDIXON-Quant imaging has high reliability in measuring fat content in lumbar bone marrow and paravertebral muscle, which is suitable for clinical use. However, quantitative measurements of fat content in paravertebral muscles using the mDIXON-Quant sequence have not been reported. In addition, in order to ensure the reliability of the results, we used ICC to evaluate the consistency of the measurements within and between observers. The correlation coefficients were all>0.75, indicating that the measurement results were consistent and reproducible.

The relationship between muscle atrophy and fatty infiltration due to degeneration of the cervical paracervical muscles and the degree of cervical disc degeneration is unclear. There was no statistical difference between the degree of disc degeneration and CSA (P>0.05), or between the degree of disc degeneration and CSA (P>0.05). The possible reason is that muscle morphology and CSA are affected by many factors, and when CSA does not change, FI of neck muscle has increased, and CSA of paravertebral muscle is not correlated with FI (28). FI was more pronounced in MF and erector spinae in patients with severe disc degeneration (29). Cloney et al. (30) found that FI of cervical MF was associated with decreased sensation and function in CSM patients. Meanwhile, extensive FI was observed in the cervical extensors of patients with chronic neck pain-related diseases, but the maximum FI was observed in the deep neck extensors such as MF and SCer compared with the superficial tissues (such as SCap, SPL) (31). Our study found that there was a difference in FI of paracervical muscles among patients with disc degeneration degree, and multiple regression analysis showed that disc degeneration degree was an independent factor affecting FI of paracervical muscles, indicating that it was not affected by other factors. Further Spearman correlation analysis showed that the degree of disc degeneration was moderately or strongly correlated with FI, and the degree of disc degeneration at C5–6 was most closely correlated with FI of deep cervical extensor (MF+SCer), showing a strong correlation. Among the cervical paravertebral muscles, the deep cervical extensor group, dominated by MF and SCer, attaches directly to the cervical spine and is considered to play a key role in maintaining stability and biomechanics, and these muscles may be sensitive to changes in neck function and pain (31). It may be related to the distribution, morphology and density of muscle spindles of different muscles in the neck, which is one of the factors for proprioceptive regulation of skeletal muscles (32). It has been found that the relative CSA of the most superficial extensors of the neck (SCap, SPL) is not reduced compared to the rectus capitis posterior minor, because muscles with high muscle spindle density (e.g., rectus capitis posterior minor and rectus capitis posterior major) may be more sensitive than those with low spindle density (SCap, SPL) (33). Our results show that the more severe the disc degeneration, the more likely the fat infiltration of the deep neck extensors (MF+SCer) is, similar to the results previously reported in patients with low back pain, and muscle degeneration is more common in lumbar MF.

## Limitations

There are a few limitations of the present study. Our study only selected C5–6, the most common cervical disc degeneration, and did not include more segments (such as C4–5 and C6–7) for comparative study. The Pfirrmann classification system for evaluating disc degeneration in this study is not perfect, and other signs of disc degeneration such as spinal cord compression, disc herniation, intervertebral foraminal stenosis, Schmermer’s node, etc. cannot be considered. It is impossible to accurately identify and isolate individual muscles in the deep neck extensors due to MRI images. In the future, it is hoped that higher resolution images and more advanced MRI sequences will better identify and differentiate individual muscles in the cervical paravertebral muscle group.

## Conclusions

There is a correlation between disc degeneration and paravertebral muscle degeneration, especially in deep neck extensors (MF and SCer). Therefore, patients with cervical disc degeneration should exercise paraspinal muscles more actively in the early stage, which is helpful to restore paraspinal muscle degeneration function and delay degeneration process.

## Data availability statement

The raw data supporting the conclusions of this article will be made available by the authors, without undue reservation.

## Ethics statement

The studies involving humans were approved by ethics committee of the People’s Hospital of Ningxia Hui Autonomous Region. The studies were conducted in accordance with the local legislation and institutional requirements. The participants provided their written informed consent to participate in this study.

## Author contributions

QL: Conceptualization, Data curation, Formal analysis, Methodology, Software, Writing – original draft, Writing – review & editing. XL: Data curation, Writing – original draft, Writing – review & editing. RW: Conceptualization, Data curation, Software, Writing – review & editing. PN: Conceptualization, Methodology, Software, Writing – review & editing. LC: Conceptualization, Methodology, Software, Supervision, Writing – review & editing. LW: Conceptualization, Funding acquisition, Methodology, Supervision, Visualization, Writing – review & editing. YS: Conceptualization, Funding acquisition, Methodology, Supervision, Writing – review & editing.
