# Corrigendum: Correlation between degeneration of cervical intervertebral disc degeneration of paravertebral muscle

**DOI:** 10.3389/fendo.2024.1454208

**Published:** 2024-07-30

**Authors:** Qiujiang Li, Xingxia Long, Rui Wang, Pengying Niu, Lijun Cai, Lei Wang, Yueming Song

**Affiliations:** ^1^ Department of Orthopaedics, Orthopaedic Research Institute, West China Hospital, Sichuan University, Chengdu, China; ^2^ Department of Thoracic Surgery, West China Hospital, Sichuan University/West China School of Nursing, Sichuan University, Chengdu, China; ^3^ Medical Center, People’s Hospital of Ningxia Hui Autonomous Region, Yinchuan, Ningxia, China; ^4^ Department of Orthopedics, People’s Hospital of Ningxia Hui Autonomous Region, Yinchuan, Ningxia, China

**Keywords:** paraspinal muscle degeneration, intervertebral disc degeneration, mDIXON-Quant, fat infiltration, cervical

**Change of authorship**

In published article, co-first shared authorship was omitted for “[Qiujiang Li and Xingxia Long]”, and first and last name were swapped for co-author Pengying Niu.

**Incorrect Affiliations**

In the published article, there was an error in the affiliations. The correct affiliations and author list appears below:

Qiujiang Li1,†, Xingxia Long2,†, Rui Wang3, Pengying Niu 3, Lijun Cai4,*, Lei Wang1, Yueming Song1

1. Department of Orthopaedics, Orthopaedic Research Institute, West China Hospital, Sichuan University, Chengdu, China.

2. Department of Thoracic Surgery, West China Hospital, Sichuan University/West China School of Nursing, Sichuan University, Chengdu, China.

3. Medical Imaging Center, People’s Hospital of Ningxia Hui Autonomous Region, Yinchuan, Ningxia, China.

4. Department of Orthopedics, People’s Hospital of Ningxia Hui Autonomous Region, Yinchuan, Ningxia, China.

The authors apologize for this error and state that this does not change the scientific conclusions of the article in any way. The original article has been updated.

